# *Salmonella* serovars associated with Grenadian tree boa (*Corallus grenadensis*) and their antimicrobial susceptibility

**DOI:** 10.1002/vms3.234

**Published:** 2020-01-14

**Authors:** Elizabeth M. Rush, Victor A. Amadi, Roger Johnson, Nicholas Lonce, Harry Hariharan

**Affiliations:** ^1^ Department of Pathobiology School of Veterinary Medicine St. George’s University St. George’s Grenada West Indies; ^2^ Antech Imaging Services Irvine CA USA; ^3^ Windward Island Research and Education Foundation Grenada West Indies; ^4^ Office International des Epizooties (OIE) Salmonella Reference Laboratory Public Health Agency of Canada National Microbiology at Guelph Guelph ON Canada

**Keywords:** antimicrobial susceptibility, *Corallus grenadensis*, Grenadian tree boa, *Salmonella*, serovars

## Abstract

Cloacal swabs from 45 Grenada bank tree boas (*Corallus grenadensis*) were sampled during a 12‐month period (2011–2012) from the rain forests and scrublands of Grenada. Cloacal swabs were examined by enrichment and selective culture for the presence of *Salmonella* spp. In all, 16 (35.6%) of the snakes were positive for *Salmonella*, and six serovars of *Salmonella* were isolated. The most common serovar was Rubislaw (31.3%), the most frequent serovar recently isolated from green iguanas in Grenada, followed by serovar Braenderup (18.8%), and serovar IV:48:g,z51:‐ (formerly, *S*. Marina) (18.8%), also found in green iguanas in this country. The remaining three less frequent serovars were, IV:53:g,z51:‐, I:6,7:e,h:‐ and IIIb:38:i:z. Antimicrobial susceptibility tests conducted by a disc diffusion method against amoxicillin–clavulanic acid, ampicillin, cefotaxime, ciprofloxacin, enrofloxacin, gentamicin, imipenem, nalidixic acid, streptomycin, tetracycline and trimethoprim–sulfamethoxazole showed that drug resistance is minimal, with intermediate susceptibility, only to streptomycin. This is the first report of isolation and antimicrobial susceptibilities of *Salmonella* serovars from wild Grenadian tree boas.

## Abstract 2

To understand the epidemiology of salmonellosis, it is important to know about the actual serovar prevalence in animals including wildlife.

## 1 INTRODUCTION

*Corallus grenadensis* is a non‐venomous boa species of snake found in Grenada and Carriacou, Canouan and Union Island. It is found mainly in drier scrublands and wet rainforests, and rarely found in captivity (McDiarmid, Campell, & Toure, 1999). *Corallus grenadensis* is a CITES appendix II level protected species. They play an important role of biological control of rodents because they eat rats and mice as a large portion of their prey diet. The Forestry and National Parks Department of Grenada rightly wants to protect and conserve this important and critical wildlife species in Grenada, which is not harmful to humans, but often comes in contact with humans, crop farms (nutmeg, cocoa, etc.) and livestock (poultry, swine, etc.). The population is being studied and monitored in multiple locations throughout Grenada, and in the dry and rainforests recovering from the passage of Hurricane Ivan in 2004. It is illegal to capture these animals without permission of the Ministry of Forestry in Grenada, and illegal to maintain this species in captivity (Forestry & Grenada, 2^2011^). Reptiles, including snakes, are considered to be a source of *Salmonella* infection for humans, but little is known about the actual serovar prevalence in healthy snakes (Goupil et al., 2012). Snake‐associated salmonellosis occurs to a considerable extent in the United States (Whitten, Bender, Smith, Leano, & Scheftel, 2015). Other recent studies include characterization of *Salmonella* isolated from captive snakes in Croatia (Lukac, Pedersen, & Prukner‐Radovic, 2015), captive and free‐living snakes in Germany (Krautwald‐Junghanns, Stenkat, Szabo, Ortlieb, & Blindow, 2013), wild snakes in Japan (Kuroki, Ishihara, Furukawa, Okatani, & Kato, 2013), wild snakes in French Guiana (Gay, Hello, Weill, Thoisy, & Berger, 2014) and python snakes kept as pets in Italy (Dipineto et al., 2014).

Recent studies in Grenada have shown that wildlife, consisting of cane toads, green iguanas, blue land crabs and small Indian mongooses are reservoirs of a variety of *Salmonella* serotypes (Drake, Amadi, Zieger, Johnson, & Hariharan, 2013; Miller et al., 2014; Peterson et al., 2013; Sylvester et al., 2014). In view of the snake‐associated salmonellosis in other parts of the world, and considering the prevalence of S*almonella* in Grenadian wildlife, this study was undertaken. The first objective of this study was to determine the occurrence of *Salmonella* in the Grenadian tree boa, and to identify the serovars present. The second objective was to determine the resistance profiles of the isolated salmonellae against 12 antimicrobial drugs, including those used in treatment of non‐typhoid *Salmonella* infections in humans.

## 2 MATERIALS AND METHODS

The snakes were sampled from 2011 and 2012 during collection of data for a project on physical and haematological parameters on the Grenadian tree boa (currently expanded to include epidemiological, genetic and population dynamics data and sample collection) with the approval of St. George's University Institutional Animal Care and Use Committee (IACUC #10015‐R). Samples were collected from all parishes in Grenada, and during the wet and dry seasons. The snakes were gently removed from trees (using a custom extendable snake hook, Animal Equipment by Stoney LLC, Bacliff, TX, USA and Fiberglass telescoping 26’ pole, Electriduct Inc., Pompano Beach), where they are safely accessible when located in the field. Faecal swabs were obtained by gentle insertion of transport swabs (5.5″ sterile mini‐tip swabs, Puritan Medical Products) into the cloaca and distal colon. For each sampled snake, the gender, estimated age (i.e. adult or juveniles) and date of sampling were recorded.

Established culture methods were used for *Salmonella* isolation (Gorski et al., 2011) with modified enrichment methods as described by Amadi et al. (2018), Drake et al. (2013) and Sylvester et al. (2014). The cloacal swabs were placed in 10 ml of tryptic soy broth for incubation before inoculation into selective media. To increase the possibility of identifying more than one serovar in a sample, up to three red colonies with black centre from Xylosine Lysine Deoxycholate (XLD) Agar (Difco/BD) were subcultured for single colonies onto tryptic soy agar. After incubation, multiple isolated colonies on each plate were tested for agglutination with *Salmonella* O antiserum poly A‐I and Vi (Difco/BD). All isolates resembling *Salmonella*, which gave positive agglutination, were inoculated into API‐20E® (Analytical profile Index; Bio‐Merieux Inc.) strips, and incubated at 37°C for 24 hr for confirmation as *Salmonella* spp. Identified pure *Salmonella* cultures were stored in 10% sterile skim milk and shipped via Fedex in cold‐packed containers for serotyping. Reference strain of *S*. Typhimurium ATCC 14028 was used as a quality control. The *Salmonella* cultures were serotyped by established methods (Ewing, 1986; Shipp & Rowe, 1980) at the Office International de Epizooties (OIE) Reference Laboratory for Salmonellosis of the Laboratory for Foodborne Zoonoses, Public Health Agency of Canada in Guelph Ontario, Canada. The serotypes were named according to the antigenic formulae listed by Grimont (2007).

The antimicrobial susceptibility tests were carried out using the disc diffusion method as recommended by the Clinical and Laboratory Standards Institute (CLSI) using Mueller Hinton agar, and the inhibition zone sizes were interpreted as per CLSI guidelines (CLSI, 2015). The antibiotic discs used were ampicillin, amoxicillin/clavulanic acid, cefotaxime, ceftazidime, ciprofloxacin, enrofloxacin, gentamicin, imipenem, nalidixic acid, streptomycin, tetracycline and trimethoprim/sulfamethoxazole (Becton, Dickinson and Co., Sparks, MD, USA). The inhibition zone sizes were interpreted based on CLSI guidelines. *Escherichia coli* ATCC 25,922 was used as quality control strain (Eguale et al., 2015).

## 3 RESULTS

A total of 45 snakes were enrolled in the study. They comprised of 32 adults (20 females and 12 males), and 13 juveniles (one female, two males, and 10 unknown gender). *Salmonella* spp. were isolated from the faecal samples of 16 (35.6%) of the 45 tested snakes. These 16 *Salmonella*‐positive snakes included nine (45%) of 20 adult females, four (33.3%) of 12 adult males and three (30%) of 10 juvenile unknown gender snakes.

Selection of up to three colonies with typical *Salmonella* morphology from each of the 16 *Salmonella*‐positive samples led to a total of 43 confirmed *Salmonella* isolates: one from one sample, two from three samples and three from 12 samples. On serotyping, these 43 *Salmonella* isolates belonged to six *Salmonella enterica* subsp *enterica* serovars: Rubislaw (12/43, 27.9%), Braenderup (9/43, 20.9%), IV:48:g,z51:‐ (9/43, 20.9%), IV:53:g,z51:‐ (6/43, 14%), I:6,7:e,h:‐(4/43, 9.3%) and IIIb:38:i:z (3/43, 7%).

Of the 16 *Salmonella‐*positive snakes, five (31.3%) yielded serovar Rubislaw which was the predominant serovar isolated. The next most frequent serovars were as follows: Braenderup (18.8%), and IV:48:g,z51:‐ (18.8%), followed by IV:53:g,x51:‐ (12.5%), I:6,7:e,h:‐ (12.5%) and IIIb:38:i:z (6.3%). Only two out of the 16 *Salmonella*‐positive snakes yielded more than one serovar. These were Braenderup + I:6,7:e,h:‐, and IV:48:g,z51 + I:6,7:e,h:‐ (Table 1).

**Table 1:** *Salmonella* serotypes isolated from 16^a^ snakes of Grenadian tree boa species

*Salmonella* serotype	Number of snakes positive	% positive
Rubislaw	5	31.3
Braenderup	3	18.8
IV:48:g,z51:‐	3	18.8
IV:53:g,z51:‐	2	12.5
I:6,7:e,h:‐	2	12.5
IIIb:38:i:z	1	6.3

a 14 snakes yielded only a single serovar, and two snakes yielded two serovars: Braenderup+I:6,7:e,h:‐, and IV:48:g,z51+I:6,7:e,h:‐

Based on results of antimicrobial susceptibility testing by the Kirby‐Bauer assay, all the 43 *Salmonella* isolates were susceptible to all antimicrobial drugs tested against, except for streptomycin, with an intermediate susceptibility rate of 46.5% (Table 2).

**Table 2:** Antimicrobial susceptibility profiles of the 43 *Salmonella* isolates recovered from snakes in Grenada

Antimicrobial (Disk conc.^a^ (µg))	Resistant	Intermediate	Susceptible
	# (%)^b^		
Ampicillin (10)	0	0	43 (100)
Amoxicillin–clavulanic Acid (20, 10)	0	0	43 (100)
Cefotaxime (30)	0	0	43 (100)
Ceftazidime (30)	0	0	43 (100)
Ciprofloxacin (5)	0	0	43 (100)
Enrofloxacin (5)	0	0	43 (100)
Gentamicin (10)	0	0	43 (100)
Imipenem (10)	0	0	43 (100)
Nalidixic acid (30)	0	0	43 (100)
Streptomycin (10)	0	20 (46.5)	23 (53.5)
Tetracycline (30)	0	0	43 (100)
Trimethoprim–sulfamethoxazole (1.25, 23.75)	0	0	43 (100)

a Resistant, intermediate or susceptible according to CLSI (2015) guideline for all drugs.

b #: number, % (percentage): values are rounded up and down to one decimal place.

## 4 DISCUSSION

Both free‐living and captive snakes are reservoirs of *Salmonella* in various parts of the world, including Taiwan (Chen et al., 2010), the United States (Goupil et al., 2012), Germany (Krautwald‐Junghanns et al., 2013), Japan (Kuroki et al., 2013), French Guiana (Gay et al., 2014). However, the results of the present study are not comparable with those from other parts of the world because of the fact that the snake species and the methods used for isolation of *Salmonella* vary. As Gay et al. (2014) noted, due to intermittent shedding of *Salmonella* in the faeces, cloacal swabbing is less sensitive than using digestive tracts from euthanized animals. The snakes used in our study, as well as some other studies, are protected by regulations, and collection of internal organs was not possible.

In the present study, of the 45 tree boa snakes, 16 (35.6%) were positive for *Salmonella*. The prevalence rates in other studies were, 58.6% in a Japanese study (Kuroki et al., 2013) of a total of 87 wild snakes of six species, and 69.7% in Taiwan in a study on 33 captive snakes of unknown species (Chen et al., 2010).

The most common serovar in our study was *S*. Rubislaw, which was isolated from five of 16 snakes. This was also the most common serovar found in green iguanas in Grenada (Sylvester et al., 2014). *S*. Rubislaw was one of the two most commonly isolated serovars from cane toads, and isolated from mongooses as well in this country (Drake et al., 2013; Miller et al., 2014). *S*. Rubislaw has also been isolated in Trinidad from *Noctilio leporinus*, a fish‐eating bat (Adesiyun, Stewart‐Johnson, & Thompson, 2009), and from wild‐caught kangaroos *(Macropus fuliginosus*) in Australia (Potter, Reid, & Fenwick, 2011). Between nine and 19 human cases of *S*. Rubislaw were reported annually in Australia from 2000 to 2009 (Moffatt et al., 2010). A Canadian study reported isolation of *S*. Rubislaw associated with human salmonellosis from an iguana and water from a turtle environment (Woodward, Khakria, & Johnson, 1997). Different serotypes of salmonellae potentially pathogenic to humans, including *S*. Rubislaw can be present in several turtle species, even within supposedly pristine environments (Gaertner, Hahn, Rose, & Forstner, 2008). An iguana‐associated case of sepsis and meningitis in a 5‐month‐old girl, due to this serovar occurred in the United States (MMWR, 1995).

*S.* Braenderup, was isolated from three snakes in the present study. *S*. Braendurup was one of the five most frequently isolated serovars from the faeces of 56 *Salmonella*‐positive Pennsylvania raccoons (*Procyon lotor*) in the United States (Very et al., 2016). Among *Salmonella* isolates from clinical non‐human sources in 2011, *S*. Braenderup was very common in horses (54% of a total of 37 isolates from all sources, submitted to the National Veterinary Services Laboratories, USA (CDC, 2011). Recently, several cases of human salmonellosis in the United States, due to serotype Braenderup were traced to a mail‐order poultry hatchery (Nakao et al., 2015). It has been implicated as a cause of a gastroenteritis outbreak in 215 people in Switzerland from eating heavily contaminated meat pies (Urfer et al., 2000). Serotype Braenderup was the sole cause of a large outbreak of *Salmonella* infection that occurred in 2008 in Japan, originating from boxed lunches containing unpasteurized liquid eggs (Mizoguchi et al., 2011). This serotype was also the sole cause of multistate human salmonellosis outbreaks in 18 states associated with consumption of raw tomatoes (MMWR, 2005). Published records of isolation of *S*. Braenderup from snakes are lacking.

Serovar IV:48:g,z51:‐, formerly known as *S*. Marina, was found in 18.8% of the tree boas in this study, and it has been associated with serious illness, including sepsis in children (Mermin, Hoar, & Angulo, 1997; MMWR, 2003). This serovar was also isolated recently from green iguanas in Grenada (Sylvester et al., 2014). There is little published information on the other serovars isolated in the present study.

Reptiles, including snakes have emerged as a significant source of human salmonellosis in the recent years. Studies are required to understand epidemiology of *Salmonella* in snakes. Schroter, Speicher, Hofmann, and Roggentin (2006) demonstrated that *Salmonella* spp. could be transmitted vertically from adult snakes to their offspring. In a study on 12 snakes of seven different species in the United States, 11 were shedders of *Salmonella*, which consisted of 15 different serotypes, and some shed two or more different serotypes (Goupil et al., 2012). In our study, two snakes were positive for more than one serovar. In Japan, faeces of a garden tree boa (*Corallus hortulanus*) were positive for *S*. Kentucky (Nakadai et al., 2004). In a study conducted in Taiwan, 69.7% of 33 snakes from various sources were positive for *Salmonella* in their faeces/cloacal swabs (Chen et al., 2010), but none belonged to the serovars found in our study.

Studies on antimicrobial drug resistance of *Salmonella* from snakes are few. None of the snake isolates in a study by Chen et al. (2010) were resistant to ciprofloxacin or other quinolone drugs. Resistance was most common to streptomycin and tetracycline. In comparison, our study showed susceptibility of all isolates to quinolones and other drugs except for streptomycin, with intermediate susceptibility of 46.5% of isolates. The previous studies on *Salmonella* isolates from green iguanas in Grenada showed intermediate susceptibility to streptomycin, tetracycline and cefotaxime, the rates being 36%, 13% and 11%, respectively (Sylvester et al., 2014). Overall, drug resistance among the snake isolates in the present study is not problematic at present, in view of the fact that no resistance was seen against cefotaxime and ciprofloxacin, two drugs commonly used for treatment of non‐typhoidal salmonellosis in humans (Chang et al., 2006; Threlfall, Skinner, Graham, Ward, & Smith, 2000).

In conclusion, the present study revealed the importance of Grenadian tree boa snake as reservoirs of serovars of *Salmonella* implicated in human disease, with serovar Rubislaw predominating. Identification of *Salmonella* serovars in wildlife can provide valuable information on potential sources of infection to humans and the possible routes of transmission. This is the first report on isolation of various *Salmonella* serovars from wild Grenadian tree boas, and their drug susceptibility patterns.

## CONFLICT OF INTEREST

None to declare.
